# Interfacial Degradation Electrochemistry of Micro‐Silicon Anodes in Solid‐State and Liquid Electrolytes

**DOI:** 10.1002/advs.76445

**Published:** 2026-07-03

**Authors:** Seokjin Kim, Jeongwoo Kim, Jaekyung Sung

**Affiliations:** ^1^ Department of Materials Engineering and Convergence Technology Gyeongsang National University Jinju Republic of Korea; ^2^ School of Materials Science and Engineering Engineering Research Institute Gyeongsang National University Jinju Republic of Korea

## Abstract

Micro‐silicon (m‐Si) is a promising next‐generation anode material for lithium‐ion batteries (LIBs), but its practical use is limited by severe volume expansion, particle pulverization, and unstable solid electrolyte interphase (SEI) formation. Recently, solidelectrolyte (SE)‐free m‐Si electrodes in all‐solid‐state batteries (ASSBs) have attracted attention as a strategy to reduce direct contact between SEs and electrode components. This architecture can suppress parasitic interfacial reactions while maximizing the active Si content. However, whether SE‐free m‐Si electrodes can deliver sufficient electrochemical performance and interfacial stability to replace conventional LIB systems remains unclear. Here, we systematically compare the degradation behavior of m‐Si electrodes in liquid‐ and solid‐electrolyte systems by correlating electrochemical performance with interface‐level structural evolution. The results reveal distinct degradation pathways depending on the electrolyte environment. In LIBs, m‐Si undergoes continuous pulverization accompanied by repeated SEI rupture and unstable SEI growth at the particle–electrolyte interface. In contrast, ASSBs promote a film‐like transformation of m‐Si with limited interfacial reactivity, improved structural integrity, and enhanced electrochemical stability. These findings clarify the critical role of electrolyte type in governing Si anode degradation and provide design guidelines for high‐energy‐density Si‐based batteries.

## Graphical abstract

This schematic compares m‐Si degradation in liquid electrolyte and solid electrolyte systems. In liquid electrolyte, repeated cycling induces severe particle pulverization and thick, unstable SEI growth. In contrast, the solid electrolyte configuration suppresses interfacial reactions, enabling a thin, stable SEI and improved structural integrity, although partial cracking and contact loss may still occur during Si volume changes in ASSB operation.

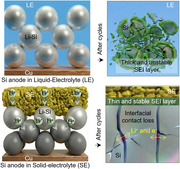

## 1 Introduction

The demand for high‐energy‐density lithium‐ion batteries (LIBs) is surging due to the rapid growth of electric vehicles and advanced energy storage systems [1, 2, 3]. In place of graphite, modified forms of silicon (Si) such as Si/C composites and SiO_x_ are being increasingly adopted to enhance anode capacity while maintaining structural stability [4, 5, 6]. However, their relatively moderate specific capacities, along with their high synthesis costs, still fall short of the requirements for next‐generation energy storage [7, 8, 9]. In contrast, the use of micro‐sized Si (m‐Si) offers a compelling alternative, not only due to its exceptionally high theoretical capacity (∼3579 mAh g^−1^) but also because of its extremely low cost, high tap density, and compatibility with established electrode fabrication techniques [10, 11, 12]. However, the use of m‐Si in conventional liquid electrolyte systems has been hindered by severe volume expansion up to 300% during lithiation, which leads to particle fracture, unstable solid electrolyte interphase (SEI) formation, and a progressive loss of electrical contact [13, 14].

To address these challenges, a growing body of research has focused on designing solid‐electrolyte‐free (SE‐free) electrode architectures for all‐solid‐state batteries (ASSBs), in which no solid electrolyte is incorporated inside the electrode composite. Recent studies have explored various SE‐free Si‐based architectures, including monolithic Si wafer electrodes, structured film or columnar Si electrodes, and nano‐ and micro‐Si particle electrodes [15, 16, 17, 18, 19, 20, 21, 22]. Especially, solid separators using sulfide‐based SEs offer high ionic conductivity (> 10^−3^ S cm^−1^) and favorable mechanical properties that can accommodate some degree of electrode expansion while preventing deep SEI penetration into m‐Si particles [23, 24, 25]. In this architecture, lithium (Li) transport occurs through the lithiation of m‐Si particles, forming a Li–Si alloy that creates both ionic and electronic pathways. This configuration suppresses uncontrolled SEI formation across particle surfaces, preserves structural integrity by limiting particle pulverization, and reduces Li consumption by minimizing repetitive SEI reconstruction [26]. Notably, recent study have demonstrated that SE‐free m‐Si electrodes can achieve superior electrochemical performance and interfacial stability not only compared to conventional liquid‐electrolyte LIBs but also relative to SE‐mixed composite electrodes, highlighting their potential as an advanced anode strategy [26, 27]. Recent studies have further expanded this strategy through prelithiation, interlayer engineering, hybrid transport pathways, and alloy‐type electrode designs, aiming to improve initial Coulombic efficiency (CE), stabilize solid–solid contact, and enhance practical cell performance [28, 29, 30, 31, 32, 33]. Moreover, when stack pressure is applied, the ductile nature of Li–Si alloys allows for mechanical compliance, further stabilizing the electrode framework [27, 34].

Given these advantages, ASSBs have emerged as a promising platform for enabling high‐capacity Si anodes. To further advance technological development, recent studies have reported that SE‐free architectures can achieve stable cycling even under reduced stack pressures, highlighting the potential of mechanical design strategies to improve cell performance [32, 35]. However, despite their potential to replace conventional LIBs using Si anodes, studies on their working mechanisms remain severely limited. In contrast to the well‐characterized behavior of Si anodes in liquid electrolytes, studies involving diffusion‐mediated m‐Si structures in SEs often lack direct comparative benchmarks for practical applications [36]. Since the prior study employed a wafer‐type Si electrode rather than a conventional composite electrode typical for LIBs, liquid electrolyte could not fully infiltrate the electrode interior. This type of study makes it difficult to clarify the distinct differences between the case of an SE‐free electrode, where all Li pathways depend on Si within the electrode, and the case of a liquid‐electrolyte‐based electrode, where Li pathways are provided by a liquid electrolyte [27]. Without a systematic investigation that evaluates mechanical, electrochemical, and interfacial responses across both electrolyte types, it is difficult to determine whether SEs truly provide a superior environment or merely a different one for m‐Si operation. Therefore, bridging this knowledge gap is vital for rational Si anode‐electrolyte design, especially as next‐generation batteries increasingly rely on solid‐state architectures where interfacial stability governs long‐term performance and cycle life.

In this study, we comparatively analyzed the electrochemical and mechanical performance, Li‐ion and electron transport behavior, and degradation mechanisms of SE‐free m‐Si electrode in ASSB (Si‐S) system. Further, it was compared with m‐Si electrode in LIB (Si‐L), where all of the particles are contacted with liquid‐electrolyte, to investigate the behavior of Li‐ion pathways and its interfacial reactions. As a result, the Si‐L exhibited continuous pulverization of Si particles and side reactions with the liquid electrolyte, which led to an increase in SEI resistance (R_SEI_), resulting in poor electrochemical performance and severe electrode thickness expansion (∼60 µm). In contrast, the Si‐S exhibited film‐like transformation of Si and a limited contact area between the electrolyte and Si, which contributed to a more stable R_SEI_, improved electrochemical performance, and reduced electrode thickness change (∼40 µm). Notably, under high‐rate conditions, the Si‐L shows a pronounced increase in overpotential due to the intrinsic two‐phase‐like reaction mechanism of Si, which limits Li‐ion and electron transport pathways. In contrast, the Si‐S follows a one‐phase‐like reaction mechanism, enabling more stable reactions without significant overpotential rise. In addition, in aging and voltage holding tests, Si‐L requires a longer time to reach steady‐state current and exhibits poor interfacial stability, higher interfacial resistance (R_CT_), and a sharp decline in charge capacity retention compared to Si‐S. These results indicate that the SE‐free m‐Si electrode delivers superior electrochemical performance and interfacial stability in ASSBs relative to LIBs, highlighting its potential for practical solid‐state battery applications. Overall, this study provides valuable insights into the distinct degradation mechanisms of Si anodes in liquid versus solid electrolytes, offering a diagnostic framework for assessing their degradation tendencies in high‐energy‐density batteries.

## 2 Results and Discussion

### 2.1 Electrochemical Behavior and Reaction Pathway

**FIGURE 1:**
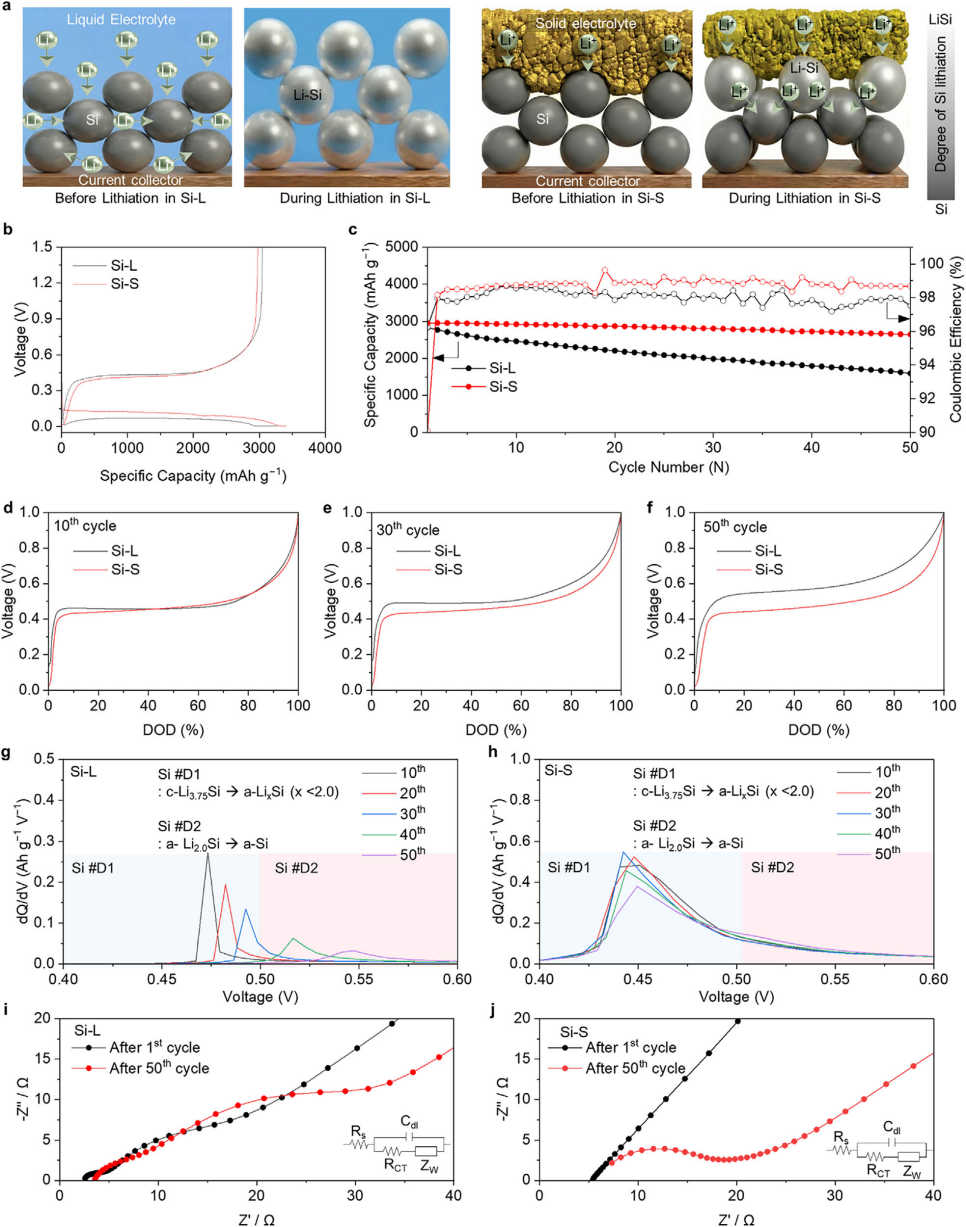
Properties of Si electrode with different electrolytes. (a) Schematic images of Si electrode during lithiation. Electrochemical performance of Si electrode with different electrolytes. (b) Voltage profile after formation. (c) Cycle performance. (d–f) Normalized voltage profile after 10th, 30th, and 50th cycle. (g, h) dQ/dV plot at 10th, 20th, 30th, 40th, and 50th cycles, respectively. (i, j) Nyquist plot after 1st and 50th cycle, respectively.

To evaluate and analyze the Si anode without hindrance of the Li‐ion pathway through other factors, we fabricated the electrodes without conventional binders, which typically act as resistive components for Li‐ion and electron transport. Instead, carbon nanotubes (CNTs) were employed as conductive additives that also serve as a binder‐like framework by forming an integrated electronic network with m‐Si particles, as confirmed by scanning electron microscopy (SEM) (Figure S1). Using the prepared electrodes, we assembled two types of cells: Si‐L and Si‐S. In the Si‐L configuration, the liquid electrolyte freely infiltrates the electrode, enabling uniform Li‐ion diffusion pathways throughout the entire electrode and promoting relatively homogeneous lithiation across all Si particles (Figure 1a). In contrast, the Si‐S configuration restricts electrolyte infiltration due to the solid nature of the electrolyte, resulting in localized lithiation beginning at Si particles directly in contact with the electrolyte. The lithiation front then gradually propagates to surrounding particles through Li‐ion diffusion by forming the Li–Si. To ensure identical conditions apart from the behavior of the Li‐ion pathway, we attempted to evaluate Si‐L under high‐pressure conditions. However, high pressure induced short‐circuiting due to the thin separator, while also hindering electrolyte penetration into the electrode (Figure S2). Thus, evaluating Si‐L under high pressure would artificially resemble the Si‐S configuration, which does not provide a meaningful distinction between the two systems. In addition, although we initially tested both configurations at the same temperature, full utilization of Si could not be achieved under those identical conditions, making it difficult to establish a clear (de)lithiation mechanism based solely on the Li‐ion pathway. Accordingly, the Si‐S cells were evaluated at 60 °C to minimize the influence of solid‐electrolyte interfacial contact limitations and to more clearly capture the intrinsic (de)lithiation behavior of Si under a sufficiently activated Li‐ion transport environment. For Si‐L, evaluations were conducted at both 25 °C (RT) and 60 °C (HT); however, Si‐L exhibited a lower average CE during cycling at 60 °C (Figure S3), indicating increased irreversible loss under this condition. This is likely associated with accelerated electrolyte decomposition and parasitic side reactions at elevated temperature, which obscure the intrinsic (de)lithiation behavior of Si. By contrast, Si‐S evaluated at 25 °C showed an ∼8% lower initial Coulombic efficiency (ICE), indicating that effective Si utilization was limited by insufficient solid‐solid interfacial contact and sluggish Li‐ion transport (Figure S4). In addition, when Si‐S was evaluated at 60°C under a reduced stack pressure of 5 MPa (low pressure, L.P), the cell exhibited inferior cycling behavior compared with that operated under 65 MPa (high pressure, H.P), further confirming that sufficient stack pressure is required to maintain solid–solid contact and continuous Li–Si transport pathways in the SE‐free electrode (Figure S5). Therefore, Si‐L and Si‐S were evaluated under their respective optimized conditions: 25 °C for Si‐L to suppress thermally accelerated side reactions, and 60 °C with sufficient stack pressure for Si‐S to alleviate transport/contact limitations. This approach enables a more meaningful mechanistic comparison of electrolyte‐dependent m‐Si degradation than enforcing identical but non‐representative test conditions. These results suggest that enforcing identical test conditions does not necessarily lead to a fair mechanistic comparison, because each electrolyte system has different transport and wetting characteristics that govern the “effective” accessibility of Si. Therefore, to probe the Si (de)lithiation mechanism without being dominated by extrinsic limitations (electrolyte infiltration/contact and transport activation), each cell was evaluated in half‐cell configurations under its own optimal conditions, where Si utilization is maximized, and the Li‐ion pathway‐dependent behavior can be most clearly distinguished. The electrochemical comparison between the two cells revealed that Si‐L exhibited a higher initial charging overpotential than Si‐S, which can be attributed to limited electrolyte infiltration due to the hydrophobic nature of CNTs within the electrode (Figure 1b,c). Si‐L delivered an initial discharge capacity of 3045.4 mAh g^−1^ and an ICE of 93.1%, while Si‐S showed a slightly lower discharge capacity of 2979.1 mAh g^−1^ and ICE of 87.3%. This discrepancy can be explained by the fact that, in Si‐L, Li‐ion and electron transport pathways are maintained through the liquid electrolyte. In contrast, Si‐S inevitably suffers from restricted Li‐ion diffusion pathways and increased diffusion lengths, likely resulting in a higher amount of trapped Li. After the 50th cycle, Si‐L exhibited a capacity retention of 56.3% with a CE of 98.0%, whereas Si‐S maintained a much higher capacity retention of 89.5% and an CE of 98.6%. To further understand this difference, delithiation voltage profiles for each cycle were analyzed (Figure 1d–f). In Si‐L, the delithiation plateau around ∼0.4 V corresponding to the c‐Li_15_Si_4_ phase gradually shortened with increasing cycles, accompanied by a noticeable rise in overpotential. Differential capacity analysis (Figure 1g) revealed that the Si #D1 peak (c‐Li_3.75_Si → a‐Li_x_Si (*x* < 2.0)) became increasingly broad, and a subsequent Si #D2 peak (a‐Li_2.0_Si → a‐Si) emerged. In contrast, Si‐S retained a sharp Si #D1 peak even after the 50th cycle, suggesting stable structural integrity and more uniform reaction behavior (Figure 1h). These differences are mainly attributed to the SEI formation behavior and the structural stability of each system. In Si‐L, liquid electrolyte continuously reacts with the Si surface, leading to the repeated formation of a thick and inhomogeneous SEI layer, which obstructs Li diffusion pathways and results in non‐uniform lithiation. On the other hand, in Si‐S, SEI formation is limited to the SE–Si interface, and the application of stack pressure helps maintain good contact between Si particles, facilitating stable Li‐ion and electron transport and contributing to the overall stability of the electrode. To further investigate the interfacial degradation, electrochemical impedance spectroscopy (EIS) measurements were conducted after the 1st and 50th cycles for both systems (Figure S6). As shown in the enlarged Nyquist plots, the Si‐L electrode exhibited a distinct high‐frequency semicircle corresponding to R_SEI_ even after the 1st cycle, with an associated resistance of approximately 5.4 Ω. By the 50th cycle, this semicircle grew significantly, and the R_SEI_ increased to 8 Ω (Figure 1i). In addition, the R_CT_ showed a modest increase from 21.6 Ω after the 1st cycle to 26 Ω after the 50th cycle, indicating aggravated interfacial/charge‐transfer polarization upon cycling. In the case of the Si‐S electrode, no high‐frequency semicircle related to SEI formation was observed after the 1st cycle, and even after the 50th cycle, only a single semicircle corresponding to R_CT_ appeared, with a resistance of approximately 13.2 Ω (Figure 1j). These results suggest that in Si‐L, the repeated reformation of SEI leads to the growth of a thick and resistive interphase, causing a continuous increase in R_CT_. In contrast, Si‐S exhibits a more limited and thinner SEI, and the resistance increase is primarily attributed to internal structural degradation of the Si electrode, such as cracking and void formation. Building upon these mechanistic insights, the practical viability of both systems was further evaluated in full‐cell configurations paired with an NCM_811_ cathode (Figure S7). Consistent with the aforementioned half‐cell results, the Si‐S full‐cell exhibited more stable electrochemical performance compared to its Si‐L counterpart. Specifically, the Si‐S system delivered a higher initial discharge capacity and ICE, alongside better cycling stability. This structural and electrochemical consistency confirms that the localized SEI formation and robust interfacial stability achieved in the Si‐S system effectively translate to reliable long‐term operation in practical cell applications.

### 2.2 Rate‐Dependent Reaction Mechanism

**FIGURE 2:**
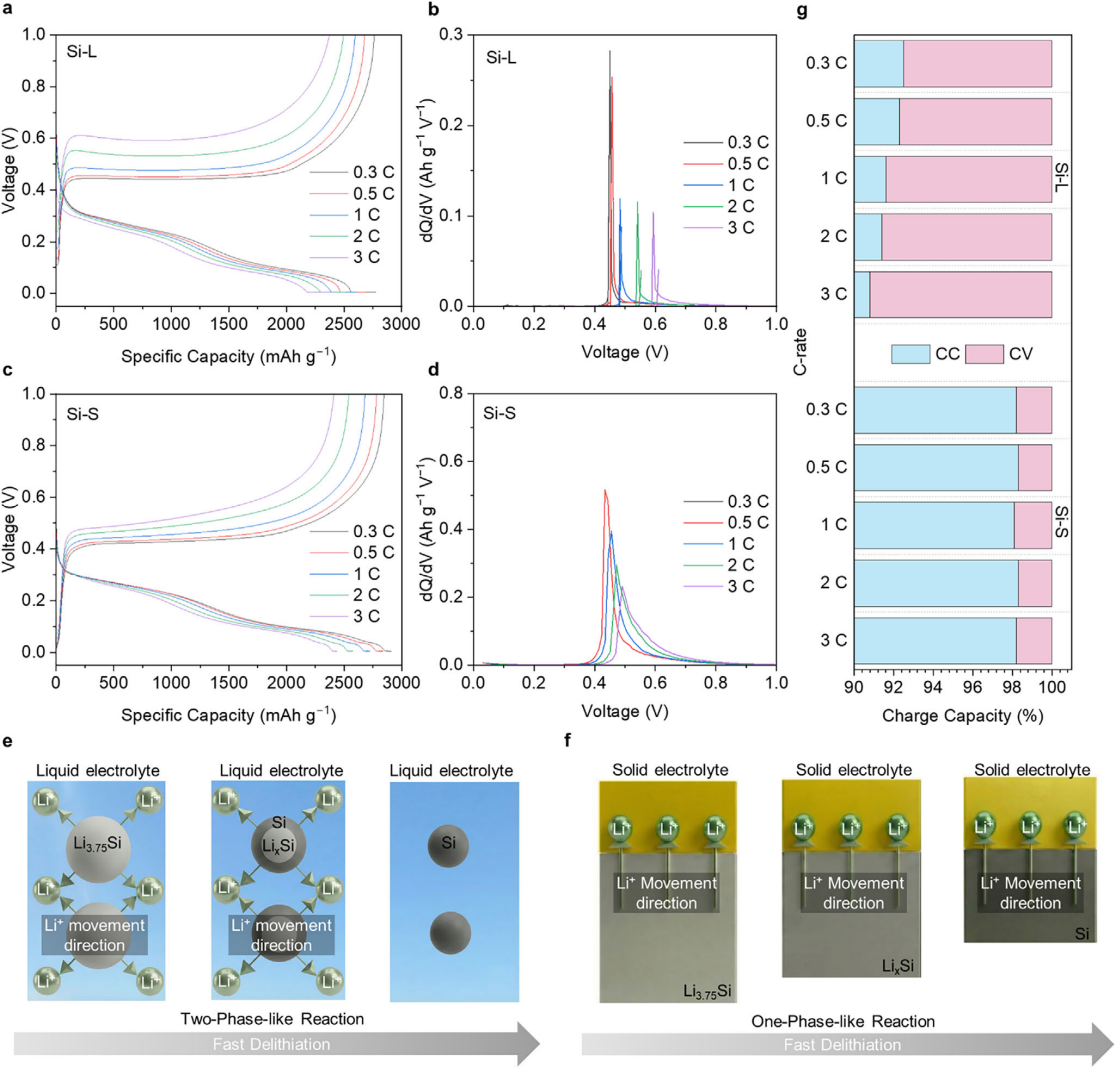
Rate capability of Si electrode with different electrolytes. (a) Voltage profile of Si‐L at different discharge current densities. (b) dQ/dV plot of Si‐L during delithiation. (c) Voltage profile of Si‐S at different discharge current densities. (d) dQ/dV plot of Si‐L during delithiation. (e, f) Schematic images of delithiation mechanism of Si‐L and Si‐S at high C‐rate, respectively. (g) Quantitative analysis of charge capacity from galvanostatic and potentiostatic steps.

To further examine the differences in Li‐ion transport and electrode reaction behavior under high‐rate conditions, rate capability tests were conducted. In the case of Si‐L, significant overpotential was observed during discharge as the current density increased, with the initial discharge voltage rising to ∼0.24 V at a rate of 3 C (Figure 2a). Corresponding dQ/dV analysis revealed that the sharp peaks gradually shifted with increasing current density due to increased overpotential, and a noticeable voltage gap of approximately 0.15 V was observed between the 0.3 and 3 C plateaus (Figure 2b). In contrast, Si‐S also showed an increase in overpotential with higher current densities, but the initial discharge voltage remained relatively stable compared to Si‐L (Figure 2c). Furthermore, while the dQ/dV peaks of Si‐S exhibited some shift at high current density, the overall sharpness of the peaks was relatively well‐maintained, indicating more stable reaction behavior even under fast cycling conditions (Figure 2d). This disparity in high‐rate behavior is attributed to the distinct delithiation pathways of the two electrodes (Figure 2e,f). In Si‐L, even under rapid delithiation conditions, the liquid electrolyte can permeate the interior of the particles, allowing delithiation to proceed from all directions. Under such conditions, a two‐phase‐like delithiation process is likely to occur, in which a relatively Li‐poor Si shell forms around a more Li‐rich Li–Si core. This phase separation results in trapped Li–Si at the core, which faces kinetic limitations for Li‐ion and electron transport due to the surrounding Si layer [37]. As a result, electrical contact within the electrode deteriorates, and localized reaction concentrations lead to increased overpotential. This manifests as a sustained discharge plateau at high rates, but with a significant increase in overpotential. On the other hand, in Si‐S, the lithiated Si is expected to behave more like a continuous Li–Si alloy film rather than discrete, phase‐separated particles, which allows more directional and spatially uniform transport of Li‐ions and electrons through the electrode. This facilitates a one‐phase‐like reaction without phase separation, maintaining good electrical contact within the electrode and enabling uniform lithiation/delithiation behavior. This interpretation is consistent with previous observations that Si in solid‐state environments can delithiate without a sharp reaction front [38]. As a result, the increase in overpotential is minimal, and while the discharge plateau shortens with increasing current density, the initial discharge voltage remains relatively stable. To further support this interpretation, ex situ X‐ray diffraction (XRD) was conducted after interrupting 3 C delithiation at a depth of discharge (DOD) of 25% (Figure S8). This DOD was selected because it corresponds to the point at which the voltage behaviors of Si‐L and Si‐S become most directly comparable: after the initial overpotential, the voltage of Si‐L begins to decrease, whereas the voltage of Si‐S increases in a relatively stable manner. Because the same Cu current collector and nearly identical Si loadings were used for both electrodes, the Cu(111) reflection was used as an internal reference for intensity normalization, allowing a semi‐quantitative comparison of the c‐Li_15_Si_4_(220) peak intensity. In Si‐L, the detection of a clear c‐Li_15_Si_4_ reflection after partial delithiation is consistent with the retention of the lithiated phase, as expected for a two‐phase‐like reaction. In contrast, a c‐Li_15_Si_4_ peak was also detected in Si‐S, although we initially expected that it would not appear. We attribute this residual c‐Li_15_Si_4_ signal to trapped Li–Si domains remaining in the SE‐free structure, where partial disconnection of Li‐ion and electron pathways can locally preserve lithiated regions even after delithiation. Nevertheless, when the normalized peak intensity was compared, the c‐Li15Si4(220)/Cu(111) intensity ratio was 1.54 for Si‐L and 0.63 for Si‐S, indicating a substantially smaller fraction of residual c‐Li_15_Si_4_ in Si‐S under the same delithiation condition. However, because ex situ XRD captures the electrode state after cell interruption and sample handling, transient non‐equilibrium Li–Si phase distributions during high‐rate delithiation may not be fully preserved. In particular, Si‐L may undergo further transformation from c‐Li_15_Si_4_ to Li‐poor Li–Si phases or amorphous Si during ex situ relaxation, whereas trapped c‐Li_15_Si_4_ in Si‐S may be retained more readily due to pathway disconnection. Therefore, the actual difference between Si‐L and Si‐S prior to ex situ sampling may be even larger than suggested by the measured intensity ratio. Thus, the ex situ XRD results provide additional structural support for different delithiation pathways in Si‐L and Si‐S. To analyze the influence of current density on Li‐ion diffusion and R_CT_ within the electrodes, the amount of charge delivered in the constant−current (CC) and constant‐voltage (CV) regions during lithiation was quantitatively compared after discharging at different current densities (Figure 2g). As a result, the CV contribution in Si‐L increased from 7.5% to 9.2% as the rate test proceeded, whereas Si‐S maintained a consistent CV contribution of approximately 1.8% across all current densities.

### 2.3 Structural Evolution of Si Electrodes

**FIGURE 3:**
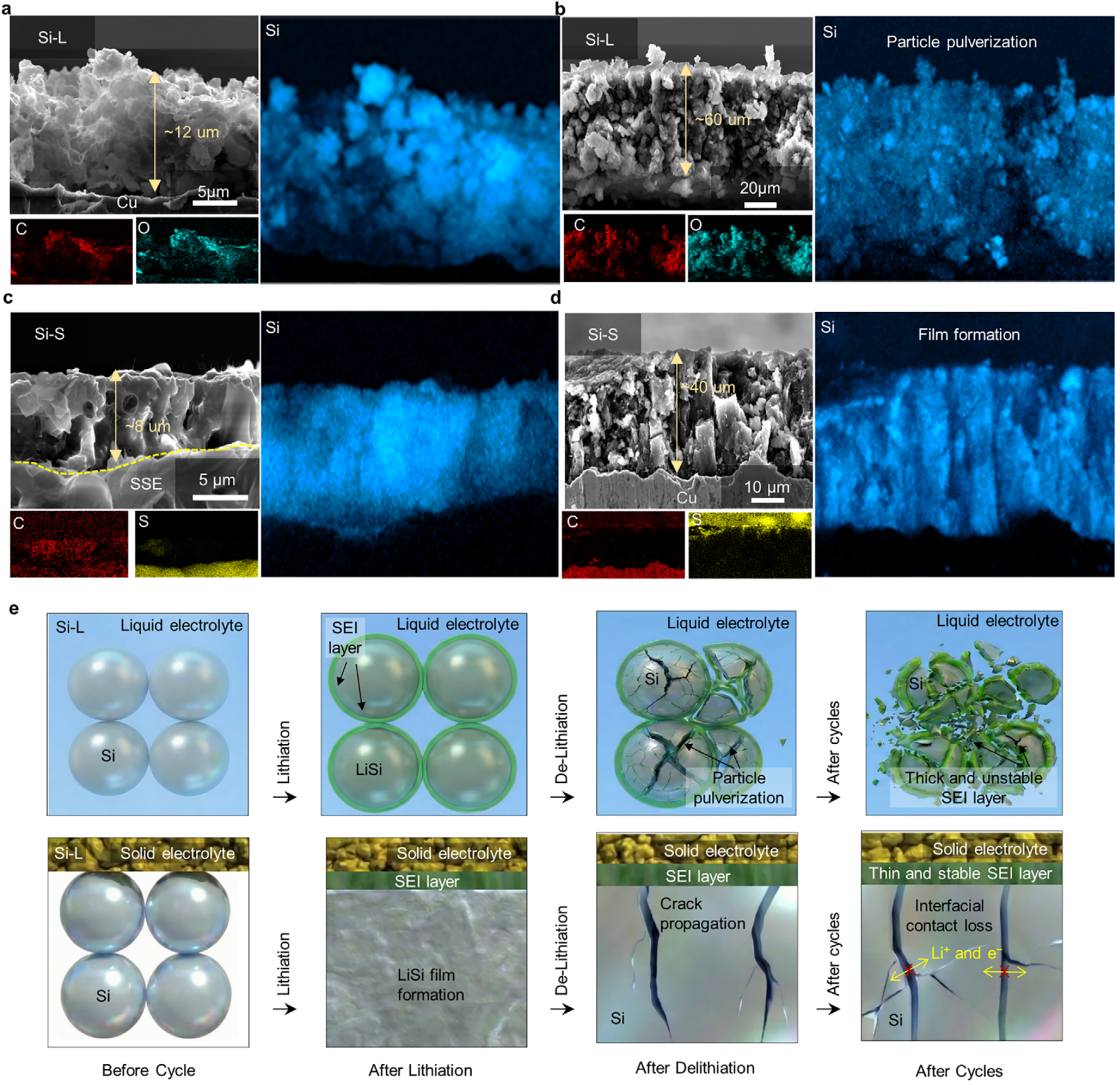
Micro‐structural analysis of Si electrode with different electrolytes. (a, b) Cross‐sectional SEM and EDS images of Si‐L after 1st and 50th cycle. c, d, Cross‐sectional SEM and EDS images of Si‐S after 1st and 50th cycles. (e) Schematic illustration of micro‐structural changes in the Si‐L and Si‐S during cycling.

For a clearer understanding of the structural degradation differences arising from the distinct degradation mechanisms in each system, microstructural changes of the electrodes after cycling were investigated. After the 1st cycle, the Si‐L electrode expanded from 7.5 to 12 µm, and energy‐dispersive spectroscopy (EDS) results confirmed that the Si particles still maintained their particulate form (Figure S9 and Figure 3a). In addition, oxygen (O) peaks were detected throughout the electrode due to the presence of major SEI components in the LIB system, such as Li_2_O and Li_2_CO_3_ [39, 40]. After the 50th cycle (Figure 3b), the electrode thickness increased up to 60 µm, with EDS analysis revealing significant degradation of the Si particles and stronger O signals. Furthermore, the cross‐sectional backscattered electron (BSE) image showed the formation of a thick surface layer with distinct contrast from the underlying Si electrode, further supporting the accumulation of a thick SEI and decomposition layer on the Si‐L electrode surface (Figure S10). In contrast, the Si‐S electrode showed minimal expansion to about 8 µm after the 1st cycle, and EDS analysis indicated transformation of Si particles into a single Si thin film (Figure 3c). After the 50th cycle (Figure 3d), the Si‐S electrode exhibited a relatively smaller thickness change (∼40 µm) compared to Si‐L, with EDS confirming that Si existed entirely as a film and columnar cracks had formed. Notably, sulfide‐related S signals were barely detected within the electrode interior, suggesting that solid‐electrolyte penetration through the formed cracks was negligible [26, 41]. Line‐scan analysis performed across the crack region further verified that sulfur was barely detectable inside the electrode, supporting the limited penetration of the solid electrolyte into the electrode interior (Figure S11). This observation is further supported by the top‐view SEM image, where the cracks generated during cycling are typically ∼1 µm in width (Figure S12a). Considering that the average particle size of LPSCl is ∼3 µm, physical penetration of the solid electrolyte through these narrow cracks is unlikely (Figure S12b). To further elucidate the chemical composition and formation mechanism of these interphases, X‐ray photoelectron spectroscopy (XPS) depth profiling was conducted for both Si‐L and Si‐S electrodes. In the case of the Si‐L electrode, the C 1s and O 1s spectra after the 1st and 50th cycles revealed liquid‐electrolyte‐derived SEI components, including organic carbonate‐related species and inorganic components such as Li_2_O, Li_2_CO_3_, and Li_x_SiO_y_ (Figure S13). These components were detected both before and after depth etching. In particular, after the 50th cycle, SEI‐related components remained clearly detectable even after depth etching, and the relative contribution of inorganic O‐containing species such as Li_2_O and Li_x_SiO_y_ became more pronounced in the etched region. This indicates that the SEI in Si‐L is not limited to the outermost surface, but develops as a thick and chemically heterogeneous interphase during cycling. Similarly, the Si‐S electrode also exhibited electrolyte‐decomposition‐derived species. The S 2p spectra after both the 1st and 50th cycles showed a low‐binding‐energy doublet assigned to Li_2_S, which is a representative inorganic SEI/interphase component formed by reductive decomposition of the sulfide solid electrolyte (Figure S14). Therefore, the interphase in Si‐S is chemically distinct from the organic/inorganic mixed SEI formed in Si‐L and should be understood as an inorganic sulfide‐derived SEI/interphase. After the 50th cycle, the Li2S contribution was observed both before and after depth etching and became more pronounced compared with that after the 1st cycle, indicating progressive LPSCl decomposition and accumulation of the inorganic sulfide‐derived interphase during cycling. However, the detection of Li_2_S does not indicate extensive solid‐electrolyte decomposition throughout the electrode. When combined with the SEM‐EDS line‐scan and EIS results (Figure 1i,j), these XPS data reveal a clear difference in the spatial distribution of interphase formation. In Si‐L, liquid‐electrolyte decomposition leads to the formation of a thick and inhomogeneous SEI throughout the electrolyte‐accessible electrode region. In contrast, in Si‐S, partial LPSCl decomposition forms a thin and relatively uniform inorganic sulfide‐derived SEI/interphase localized at the SE–Si solid–solid contact interface. The differences in outcomes between the systems can be explained by schematic illustrations (Figure 3e). In the case of Si‐L, as the Si particles undergo lithiation, the infiltrated liquid electrolyte forms an SEI layer on all interfaces. During discharge, the volume contraction of Si particles exposes new interfaces where no SEI has formed, leading to particle pulverization and the progressive development of an increasingly thick and uneven SEI layer as cycling continues. On the other hand, in Si‐S, a spatially confined interphase forms initially only at the interface contacting the solid electrolyte layer during lithiation, and the external stack pressure causes the ductile Li–Si alloy to transform into a single film. Subsequently, volume contraction of the formed film combined with vertical stack pressure induces the formation of columnar cracks. Although these cracks gradually grow with cycling, their size remains too small to allow solid electrolyte penetration, thereby limiting extensive interphase formation inside the electrode. However, the accumulated cracks can interrupt continuous Li‐ion and electron transport pathways within the Li–Si film, causing partial electrochemical isolation of Si and contributing to performance degradation in the Si‐S system.

### 2.4 Interfacial Stability and Electrolyte Decomposition Behavior

**FIGURE 4:**
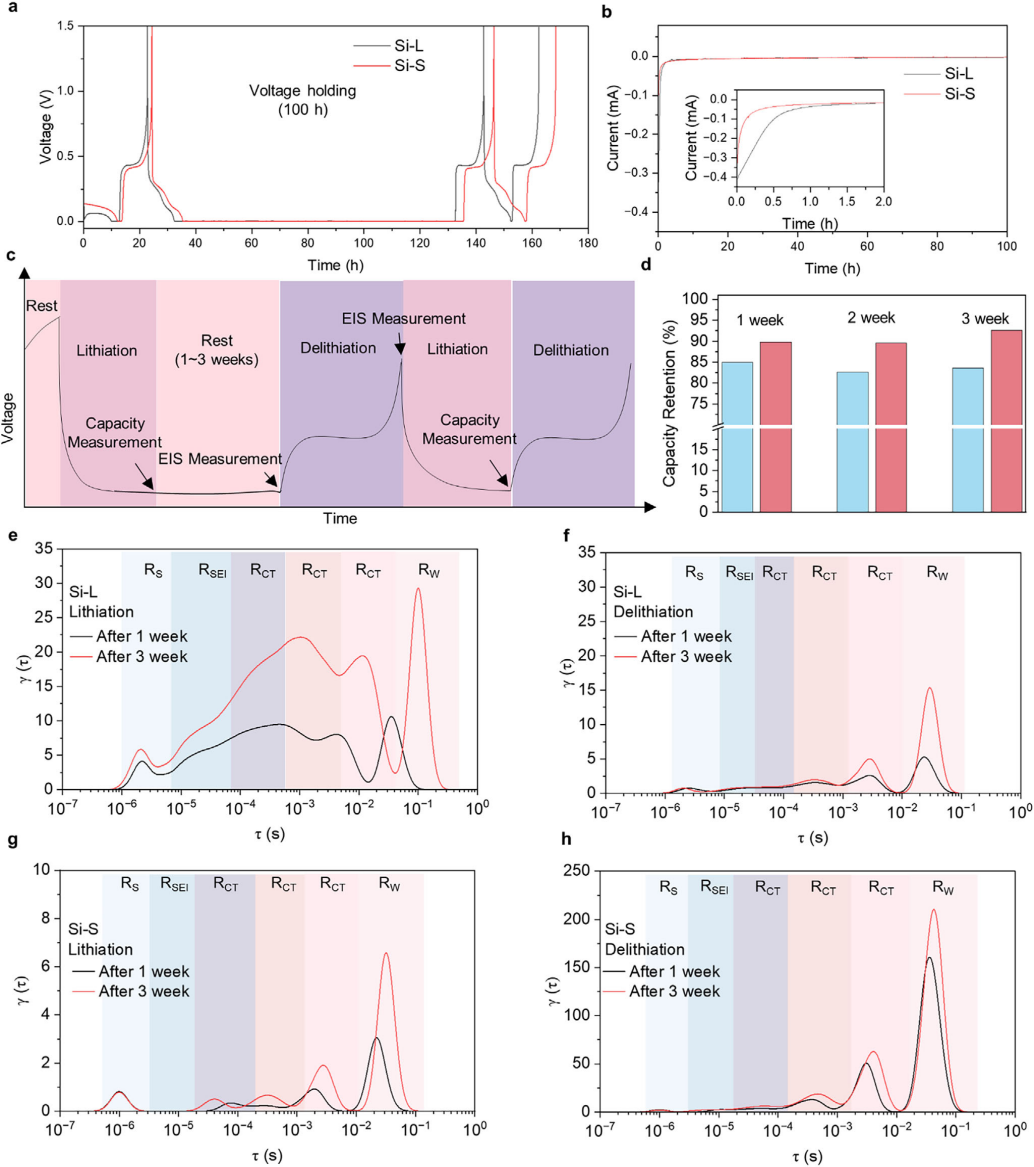
Electrochemical analysis of Interfacial reaction for Si electrode with different electrolytes. (a) Voltage profile during voltage holding test. (b) Time‐current plot during 100 h of voltage holding. (c) Graphical protocol for aging test. (d) Comparison of charge capacity after aging test. DRT analysis of Si‐L and Si‐S after 1 and 3 weeks. (e, f) Si‐L after lithiation and delithiation, respectively. (g, h) Si‐S after lithiation and delithiation, respectively.

In this way, Si‐L experiences continuous and unstable SEI layer formation due to direct exposure of Si particles to the electrolyte along with particle pulverization, ultimately causing severe performance degradation. In contrast, Si‐S shows minimal direct electrolyte exposure and maintains excellent performance thanks to a stable SEI layer. This indicates that the extent of side reactions between Si and the electrolyte plays a critical role in determining performance. To quantitatively analyze the decomposition reactions of liquid and solid electrolytes, voltage holding and aging tests were conducted. First, to examine the decomposition reaction between lithiated Si and the electrolyte, the voltage was held at 0.005 V for 100 h after formation (Figure 4a). Comparing the current changes during the holding time, Si‐S reached the steady‐state current slope faster than Si‐L (Figure 4b). This indicates that continuous side reactions occur between lithiated Si and the electrolyte in Si‐L, whereas such side reactions are significantly suppressed in Si‐S [42, 43]. Figure 4c shows the protocol for the aging test, which was performed without a pre‐formed SEI layer to enable clear comparison and analysis. The Si‐L cell exhibited low charge capacity retention of 85.0%, 82.6%, and 83.6% after 1, 2, and 3 weeks, respectively, while the Si‐S cell showed higher retention rates of 89.8%, 89.6%, and 92.6% (Figure 4d and Figure S15). Despite using the same electrode, the greater capacity loss in Si‐L is attributed to more active decomposition reactions with the liquid electrolyte during aging, which limits the active Si. On the other hand, in Si‐S, decomposition reactions are confined only to the interface with the solid electrolyte used as the separator, resulting in relatively stable Si particles and electrode structure. To analyze the measured EIS data in more detail, they were converted and analyzed using distribution of relaxation time (DRT) (Figure S16 and Figure 4e–h) [44]. After 3 weeks of lithiation, all resistances in Si‐L increased up to approximately three times compared to 1 week, resulting in an overall shift in the impedance spectrum (Figure 4e). This is interpreted as continuous decomposition reactions due to the low electrochemical stability between lithiated Si and the liquid electrolyte. EIS data measured after delithiation showed similar resistance increases across all regions, especially in the R_SEI_ region (Figure 4f and Figure S17a). In contrast, Si‐S exhibited almost no change in resistance between 1 and 3 weeks after lithiation, and no distinct peak was observed in the R_SEI_ region, indicating minimal SEI formation (Figure 4g). Delithiation results (Figure 4h and Figure S17b) also showed negligible changes in resistance, with no increase in the R_SEI_ region. This suggests that not only was interfacial reaction limited by the absence of liquid electrolyte inside the electrode, but also that the sulfide‐based SE and lithiated Si are electrochemically more stable than liquid electrolytes. However, overall resistance values were significantly higher in Si‐S than in Si‐L, which is attributed to crack formation in Si particles: in Si‐L, liquid electrolyte infiltration maintains Li‐ion pathways, whereas in Si‐S, solid electrolyte cannot penetrate, leading to electrochemical isolation and increased resistance. In addition, the Warburg impedance region was more pronounced, likely because the electrode is composed purely of diffusion‐limited m‐Si without any SE inside, which has low Li‐ion conductivity. Therefore, the SE‐free m‐Si electrode demonstrates more stable interfaces and better electrochemical performance in solid electrolyte environments than in liquid electrolyte systems. Nevertheless, the relatively high overall resistance and pronounced Warburg response in Si‐S indicate that the present SE‐free architecture still requires further optimization. Future studies should focus on reducing diffusion limitations in m‐Si electrodes by controlling particle size, electrode thickness, and the continuity of Li–Si transport pathways, while preserving the confined electrolyte‐access feature that suppresses repeated interphase growth. In addition, optimization of stack pressure and mechanically compliant conductive frameworks or interlayers could further improve solid–solid contact and mitigate crack‐induced electrochemical isolation. These directions are expected to improve the rate capability and long‐term cycling stability of SE‐free m‐Si electrodes without sacrificing the interfacial advantages identified in this study.

## 3 Conclusion

In this study, the electrochemical, structural, and interfacial properties of m‐Si electrodes were comparatively investigated in Si‐L and Si‐S systems using an identical electrode configuration. This analysis aimed to evaluate the effectiveness and practical applicability of the SE‐free m‐Si electrode in ASSBs. In the Si‐L system, severe performance degradation was observed due to the pulverization of Si particles induced by repeated volume changes during cycling, as well as the formation of thick and unstable SEI layers resulting from continuous contact with the liquid electrolyte. This led to a significant increase in R_CT_ and pronounced electrode thickening. In contrast, the Si‐S system exhibited suppressed Si pulverization due to the inability of the solid electrolyte to penetrate the electrode interior. The limited interfacial area between the solid electrolyte and Si particles enabled the formation of thin and uniform SEI layers, thereby maintaining more stable long‐term cycling performance with relatively lower resistance growth and reduced thickness change. Under high‐rate conditions, Si‐L exhibited inefficient ion and electron pathways due to its two‐phase‐like reaction mechanism, leading to a sharp increase in overpotential. Meanwhile, Si‐S promoted a one‐phase‐like reaction, ensuring more uniform reaction pathways and improved electrochemical and structural stability. Additional voltage holding and aging tests further demonstrated the differences in interfacial stability. Si‐L showed delayed stabilization of steady‐state current and a pronounced decline in charge capacity retention, attributed to unstable interfacial reactions and increasing resistance. On the other hand, Si‐S reached current stabilization more quickly and maintained relatively stable electrochemical performance and R_CT_ under aging conditions. Beyond the performance comparison, this work clarifies why the same m‐Si electrode undergoes distinct degradation pathways in liquid‐ and solid‐electrolyte environments. By identifying electrolyte accessibility, interfacial reaction area, Li–Si transport continuity, and pressure‐assisted structural evolution as key factors governing Si degradation, this study provides mechanistic design guidelines for future SE‐free Si anodes and high‐energy‐density solid‐state batteries.

## Author Contributions

**Seokjin Kim**: conceptualization, methodology, investigation, validation, formal analysis, writing – original draft. **Jeongwoo Kim**: methodology, investigation, validation. **Jaekyung Sung**: conceptualization, methodology, investigation, validation, funding acquisition, supervision, writing – review and editing.

## Conflicts of Interest

The authors declare no conflicts of interest.

## Supporting information




**Supporting File**: advs76445‐sup‐0001‐SuppMat.docx.
