# Supplementary material for: Interfacial Degradation Electrochemistry of Micro‐Silicon Anodes in Solid‐State and Liquid Electrolytes
Source: Adv Sci (Weinh). 2026 Jul 3;13(54):e76445. doi: 10.1002/advs.76445 (PMC13334594; doi:10.1002/advs.76445)
Supplement: Supplementary file 1 [file ADVS-13-e76445-s001.docx]

**Supplementary information
Interfacial Degradation Electrochemistry of Micro-Silicon Anodes in Solid-State and Liquid Electrolytes**

**Methods**

**Material preparation**

The Si particles (μ-Si, 1-10 μm) dried in vacuum oven at 80℃ overnight before use. SWCNT (H_2_O, 0.4wt% CMC) used for the Si anode was obtained from OCSIAl. Li metal chip was (Thickness : 250 μm , Φ = 13 mm and 16 mm) used as counter electrode at half-cell. In the case of Electrolytes, for Si-L, 1.3 M LiPF_6_ in ethylene carbonate (EC)/ethyl methyl carbonate (EMC)/diethyl carbonate (DEC) (3/5/2, v/v) with 10% of fluoroethylene carbonate (FEC), 0.2% of lithium tetrafluoroborate (LiBF_4_), 0.5% of vinylene carbonate (VC), 3% of succinonitrile (SN), and 1% of propane sultone (PS) were used as electrolyte for half-cell. For Si-S, Li_6_PS_5_Cl (LPSCl, D_50_ = ~3 um) was used as solid electrolytes.

**Preparation of Si anode**

To fabricate the Si anode, a slurry was prepared using 98 wt% of μ-Si and 2 wt% of SWCNT and D.I water solvent before casting on a current collector. Slurry was mixed using Thinky mixer (Thinky Corporation, AR-100). Carbon-based coated Cu foil was used as current collector and Doctor blade was using for casting on current collector. The areal capacity of the prepared electrodes was controlled to be 3.0 **±** 0.3 mAh cm⁻², corresponding to a variation within approximately **±**10%, and the same loading range was used for both Si-L and Si-S cells before electrochemical testing. For the full-cell evaluations, the areal capacity of the prepared electrodes was specifically adjusted to 2.6 **±** 0.1 mAh cm⁻².

**Preparation of NCM Cathode**

The cathode electrodes for the Si-L and Si-S systems were prepared using distinct methods. For the Si-L system, the cathode electrode was directly obtained from SMLab. The composition of this electrode consisted of 94 wt% active material, 3 wt% Carbon black, and 3 wt% Poly(vinylidene fluoride) (PVdF) binder. Conversely, the cathode for the Si-S system was internally prepared by blending NbO-coated NCM_811_ as the active material, LPSCl as the solid electrolyte, and vapor-grown carbon fibers (VGCF) as the conductive additive in a weight ratio of 70:27:3. The composite mixture was thoroughly homogenized using a mortar and pestle. For both cathode systems, aluminum (Al) foil was utilized as the current collector, and the areal capacity of the prepared electrodes was uniformly controlled to be 2.3 **±** 0.1 mAh cm⁻².

**Cell assembly**

To fabricate the Si-L, 2032 coin-type half-cell was assembled by stacking Si anode, microporous polyethylene (20 μm, Celgard), and Li metal chip (Φ = 16 mm) with 50 μl of electrolyte. For the Si-L full-cells, a cathode (Φ = 10 mm) was stacked instead of the Li metal chip, utilizing the same amount of electrolyte. To fabricate the Si-S, LPSCl powder (153 mg) was first compressed into a pelletized separator under a pressure of 100 MPa. Subsequently, the Si anode was stacked onto the separator and further compacted together at 650 MPa. After that, a Li metal chip (Φ = 13 mm) was added under 20 MPa, followed by applying overall stack pressure of 65 MPa and 5 MPa during cycling. For the Si-S full-cells, a cathode (Φ = 13 mm) was stacked instead of the Li metal. The electrodes and solid electrolyte were compacted together at 650 MPa, and the same overall stack pressure of 65 MPa was applied during cycling**.** All processes were conducted in Ar-filled glove box.

**Materials characterization**

The SEM and EDS instrument (Apreo S, Thermo) was used to investigate the surface morphology of powders and electrodes. The impedance was measured by a Biologic (VSP-300), The amplitude of the input signal was 10 mV, and the frequency range was from 1 MHz to 0.1 Hz. The cell tester was used by a WonATech (WBCS 3000)**.** The particle size distribution was measured by a particle size analyser (CILAS, 1090LD shape Analyzer). X-ray diffraction (D8 advance A25, Bruker) was used to analyze the crystal structures of particles and electrodes using Cu kα radiation with 2ϴ in the range from 20.00° to 80.00°. The chemical states of each electrodes were analysed by XPS (K-alpha, ThermoFisher).

**Electrochemical performance tests**

We applied CC/CV and CC process during charge (lithiation)/discharge (delithiation). During the formation, we conducted at a rate of 0.1C (1C = 3 mA cm^-2^) within a voltage range of 0.005 V ~ 1.5 V. For subsequent cycles, the process was performed at a rate of 0.5C with a cut-off voltage of 1 V. In the rate test, we applied the current from 0.3C to 3C each 3 cycles. At Aging test, we lithiated the Si anode up to 0.005 V and kept in 1 to 3 weeks without formation process. At voltage holding test, we first conducted the formation process and further maintained the lithiated state for 100 hours**.** For the full-cell evaluations, the initial formation step was conducted at room temperature (25 °C) in CC mode at a rate of 0.1C within a voltage range of 2.5 V to 4.2 V. For subsequent cycles, the full-cells were cycled at a rate of 1C within a voltage range of 2.8 V to 4.2 V. Prior to all electrochemical testing, the Si-L and Si-S cells were rested for 20 hours and 3 hours, respectively. The same designated charge/discharge protocols were consistently applied to both the Si-L and Si-S systems across all corresponding tests.

Fig. S1 | SEM images of electrode. a, Micro silicon. b, SWCNT. c, Si-CNT particle in the electrode.

Fig. S2 | Voltage profile of Si-L at High pressure

 Fig. S3 | Voltage profiles and cycle performance of Si-L with different temperature. a, Voltage profiles. b, Cycle performance.

Fig. S4 | Voltage profiles of Si-S with different temperature

**Fig. S5** | **Voltage profiles and cycle performance of Si-S with different stack pressure**. a, Voltage profiles. b, Cycle performances

 Fig. S6 | Nyquist plots after 1^st^ and 50^th^ cycles. a, b, Si-L and Si-S, respectively

Fig. S7 | Voltage profiles and cycle performance of Si anodes paired with NCM_811_ at room temperature. a, Voltage profiles at 0.1C. b, Cycle performances at 1C.

**Fig. S8 | Ex situ XRD analysis of residual c-Li_15_Si_4_ phase after high-rate delithiation**. **a,** Voltage profiles of Si-L and Si-S interrupted at DOD 25% during 3C delithiation. **b,** Ex situ XRD patterns of Si-L and Si-S after interruption at DOD 25%. The dashed lines indicate the characteristic reflections of **c-Li_15_Si_4_**, including the (211), (220), (310), and (332) planes, and the Cu(111) reflection from the current collector. The XRD intensities were normalized using the Cu(111) peak as an internal reference because comparable Si loading and the same Cu current collector were used.

 Fig. S9 | Cross-sectional SEM image after pressing the electrode of Si-L and Si-S..

**Fig. S10 | a**, Cross-sectional SEM and EDS images of Si-L after 50^th^ cycles. **b**, Cross-sectional BSE image of Si-L after 50^th^ cycles.

**Fig. S11 | a**, Cross-sectional SEM and EDS images of Si-S after 50^th^ cycles. **b**, Elemental line scan along yellow arrow in the Si-S electrode.

 Fig. S12 | a, Top-view SEM image of Si-S after 50^th^ cycles. b, Particle size distribution of LPSCl.

**Fig. S13 | Depth profiling XPS spectra of Si-L anodes after 1^st^ and 50^th^ cycles. a**, **b**, XPS spectra of C 1s. c, d, XPS spectra of O 1s, respectively.

**Fig. S14 | Depth profiling XPS spectra of Si-S anodes after 1^st^ and 50^th^ cycles. a**, **b**, XPS spectra of S 2p, respectively.

 Fig. S15 | Normalized voltage profile during aging test. a, b, Si-L and Si-S during lithiation, respectively.

 Fig. S16 | Nyquist plots after 1 and 3 week. a, b, Si-L and Si-S after lithiation, respectively. c, d, Si-L and Si-S after delithiation, respectively.

 Fig. S17 |. DRT analysis during delithiation. a, Si-L after 1 and 3 weeks. b, Si-S after 1 and 3 weeks.
